# On–off switching of cell cycle and melanogenesis regulation of melanocytes by non-thermal atmospheric pressure plasma-activated medium

**DOI:** 10.1038/s41598-019-50041-2

**Published:** 2019-09-16

**Authors:** Jin-Woo Lee, Se Jik Han, Hye Young Kang, Sung-Suk Wi, Min-Hyung Jung, Kyung Sook Kim

**Affiliations:** 1 Medical Science Research Institute, Kyung Hee University Medical Center, Seoul, Korea; 2 Department of Biomedical Engineering, Graduate school, Kyung Hee University, Seoul, Korea; 3 Department of Biomedical Engineering, College of Medicine, Kyung Hee University, Seoul, Korea; 4 Research Laboratory, Medipl Co., Ltd, Gyeonggi-do, Korea; 5 Department of Obstetrics & Gynecology, School of Medicine, Kyung Hee University, Kyung Hee Medical Center, Seoul, Korea; 6 Healthcare Industry Research Institute, Kyung Hee University, Seoul, Republic of Korea

**Keywords:** Biological techniques, Cell biology

## Abstract

Non-thermal atmospheric pressure (NAP) plasma has demonstrated potential in biomedical applications, such as cancer treatment, bactericidal sterilization, and cell growth promotion or inhibition. In this study, for the first time, we demonstrated on–off switching of cell cycle progression and regulated melanogenesis in normal human skin melanocytes by NAP plasma-activated medium (PAM). The melanocytes were exposed to NAP plasma at durations varying from 0 to 20 min, and the effects of PAM on cell proliferation, cell cycle progression, and melanogenesis were investigated. Although PAM showed no cytotoxicity, the proliferation of melanocytes was inhibited. The melanocyte cell cycle was arrested by PAM for a relatively short period (48 h), after which it recovered slowly. PAM promoted melanogenesis through the activation of the enzymes tyrosinase, tyrosinase-related protein-1, and tyrosinase-related protein-2. These effects seem to be related to reactive oxygen species induced by PAM. Our finding that PAM modulates the cell cycle may provide insight into the recurrence of cancer. The regulation of the melanogenesis of melanocytes may facilitate the control of skin tone without incurring negative side effects.

## Introduction

Non-thermal atmospheric pressure (NAP) plasma is emerging as a new tool in various biological and medical fields^1,2^. Because NAP plasma operates below 40 °C, it can be applied to biomaterials such as tissues or cells without a temperature increase. Plasma is a state of matter comprising ions, electrons, excited and neutral atoms, free radicals, and ultraviolet (UV) photons^3^. Reactive oxygen species (ROS) and reactive nitrogen species generated by plasma play various roles in biomaterials. Depending on the dose, NAP plasma can induce either cell proliferation or cell death. For instance, endothelial cells treated with low doses of NAP plasma showed a higher proliferation rate and released more fibroblast growth factor-2 than did untreated cells^4^, whereas a large amount of ROS generation with high doses of plasma led to DNA damage, resulting in cell cycle arrest and subsequent apoptosis^5^. A high dose of plasma can cause lipid peroxidation, transient pore formation in cell membranes, and alterations in protein structure^6^.

In the present study, we introduce NAP plasma as a potential approach to regulating single-cell functioning. We examined the effects of NAP plasma on normal human skin melanocyte functions, including proliferation, cell cycle progression, and melanogenesis. Melanocytes were exposed to plasma-activated medium (PAM), and the changes in cell number, cell cycle, melanin contents, and gene expression of tyrosinase, tyrosinase-related protein-1 (TRP-1), and tyrosinase-related protein-2 (TRP-2) were analyzed. PAM has similar effects to NAP plasma in gas and also has several additional advantages: (1) PAM can avoid any effects from UV radiation and temperature; (2) it allows for uniform treatment at a given time; and (3) it maintains a stable state for a long time under appropriate temperature conditions^7,8^. The cell culture medium was exposed to NAP plasma for 0–20 min, and the resulting PAM was applied to melanocytes. After incubating the cells in PAM for a given duration, changes in melanocyte function were observed. To our knowledge, this study is the first to demonstrate the effects of PAM in on–off switching of cell cycle progression and melanogenesis.

## Results

### Plasma-activated medium-regulated proliferation of melanocytes

**Figure 1:**
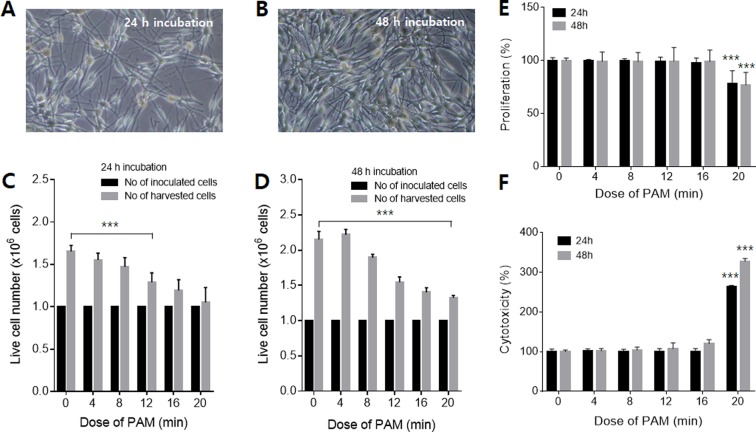
Morphological changes in melanocytes treated with PAM. The melanocytes were incubated for (**A**) 24 and (**B**) 48 h at a PAM dose of 20 min. The live cell number was counted to evaluate the effects of PAM on proliferation after (**C**) 24 and (**D**) 48 h of incubation. To confirm the cytotoxicity of PAM, (**E**) WST-1 and (**F**) LDH release assays were performed after incubation for 24 and 48 h.

Culture medium was exposed to NAP plasma for 4, 8, 12, 16, and 20 min. Because the generation of radicals was proportional to the exposure duration to NAP plasma, the exposure duration of the culture medium to plasma corresponded to the PAM dose. To investigate the effect of PAM on their viability, melanocytes were exposed to PAM for 4, 8, 12, 16, and 20 min, and subsequently incubated for 48 h. According to the tetrazolium (MTT) assay (Fig. S1), PAM seemed to have cytotoxic effects on melanocytes. However, the cell observations by optical microscopy were quite different from the MTT results. Figure 1A,B show the cells incubated for 24 and 48 h at a PAM dose of 20 min, respectively. Both groups of cells showed typical melanocyte morphology and were stably attached to the bottom. Floating or dead cells were scarce. Furthermore, the cell population did not decrease, but rather increased dramatically after 48 h. We counted the cell population directly. Figure 1C,D show the cell numbers after 24 and 48 h of incubation in PAM, respectively. In both cases, the number of cells was increased in all groups, including control and PAM-treated cells. This indicated that the melanocytes proliferated even after PAM treatment. However, the proliferation rate was lower than that for the control group, and the rate decreased as the PAM dose increased. Compared to that in the control group, cell proliferation in the group incubated in PAM for 24 h was reduced to 67%, and that in the group incubated for 48 h was reduced to 61% of the control. This result indicated that, within the observed dose and time ranges, PAM had no cytotoxic effect on melanocytes, although it inhibited proliferation.

To further evaluate the cytotoxicity of PAM, cell proliferation (WST-1) and cytotoxicity (LDH leakage) assays were performed. Exposure to PAM for 16 min did not significantly affect the proliferation rate of melanocytes after 24 and 48 h of incubation, while the proliferation rate was decreased by exposure to PAM for 20 min after both 24 and 48 h of incubation (Fig. 1E). Similarly, cytotoxicity was not influenced by exposure to PAM for 16 min but was elevated by exposure for 20 min (Fig. 1F). These findings confirmed that cells are not damaged by exposure to PAM for up to 16 min.

### On–off switching of the cell cycle by plasma-activated medium

**Figure 2:**
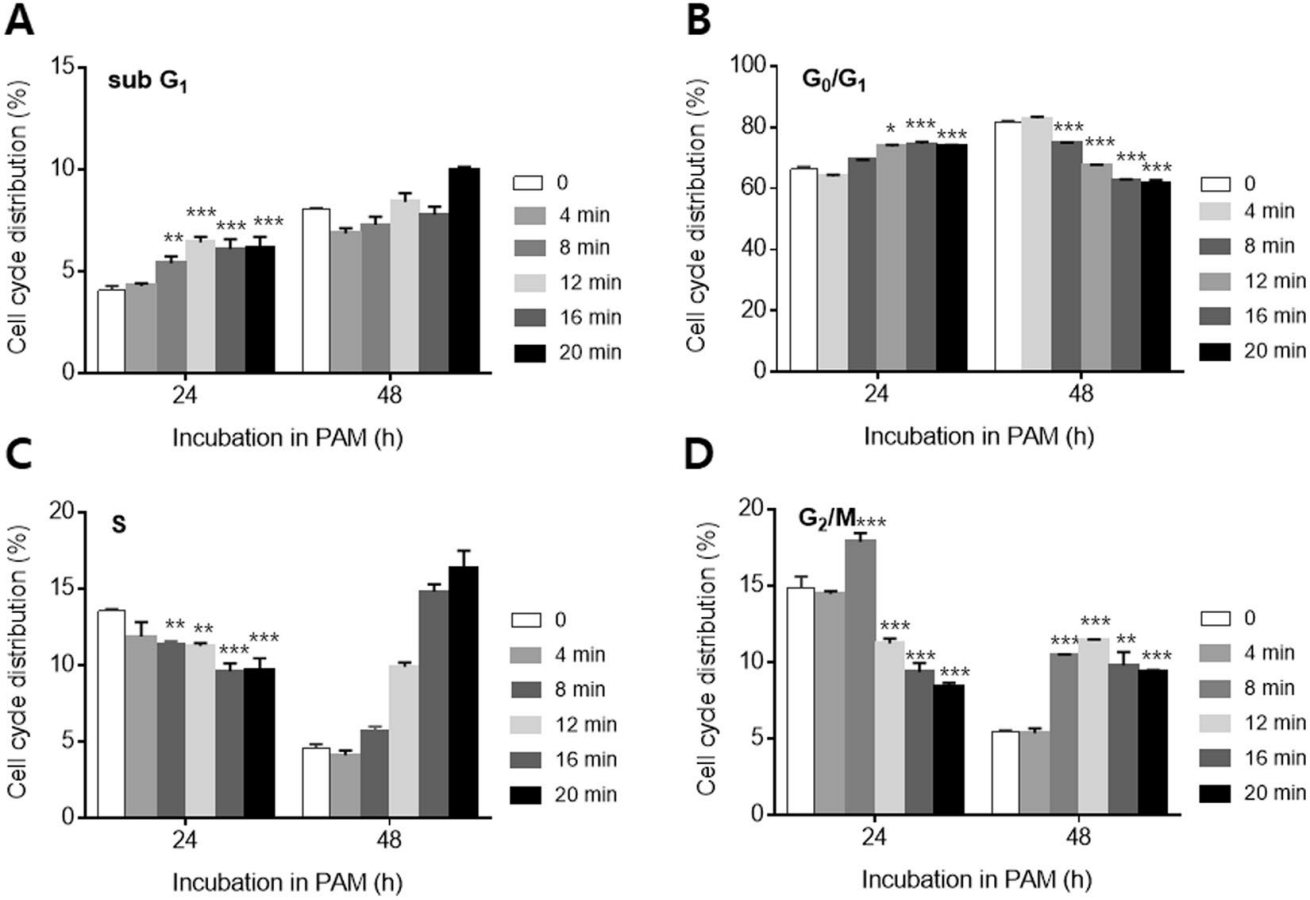
Cell cycle analysis of PAM-treated melanocytes. The cells were incubated with PAM for 24 or 48 h, and then cell cycle was determined. The dose of PAM was increased from 0 to 20 min. The distribution of cells in the (**A**) sub-G_1_, (**B**) G_0_/G_1_, (**C**) S, and (**D**) G_2_/M phases of the cell cycle were counted. The values represent the means ± SD (*p < 0.05, **p < 0.01, ***p < 0.001).

To better understand the mechanism underlying this inhibition of proliferation, flow cytometric analysis was conducted to evaluate the effect of PAM on the cell cycle distribution. After PAM exposure at varying doses ranging from 0 to 20 min, the melanocytes were incubated for 24 or 48 h. After 24 h, the sub-G_1_ phase was increased in a dose-dependent manner, and the rate of increase was statistically significant (Fig. 2A). The distribution of the G_0_/G_1_ phase also increased as the PAM dose increased (Fig. 2B). This increase in the contributions of melanocytes in the G_0_/G_1_ phase was accompanied by a concomitant reduction in the contributions of cells in the G_2_/M phase (Fig. 2D). The S phase was decreased by PAM treatment (Fig. 2C). These results indicate that the proliferation of melanocytes was inhibited through an increase in the growth phase, suppression of DNA synthesis, and mitosis. When the cells were treated for 48 h in PAM, the sub-G_1_ phase increased, but the rate of increase was not statistically significant (Fig. 2A). PAM decreased the G_0_/G_1_ phase and increased the G_2_/M phase (Fig. 2B,D). This finding indicated that the cell cycle was arrested, but the arrest was not long lasting. As the cell arrest began to diminish, DNA synthesis and mitosis increased. The S phase increased also, although not significantly. These results indicated that PAM could cause apoptosis of melanocytes; however, the level of apoptosis was not significant and lasted for only a short period. This result is consistent with the cell proliferation results shown in Fig. 1B.

**Figure 3:**
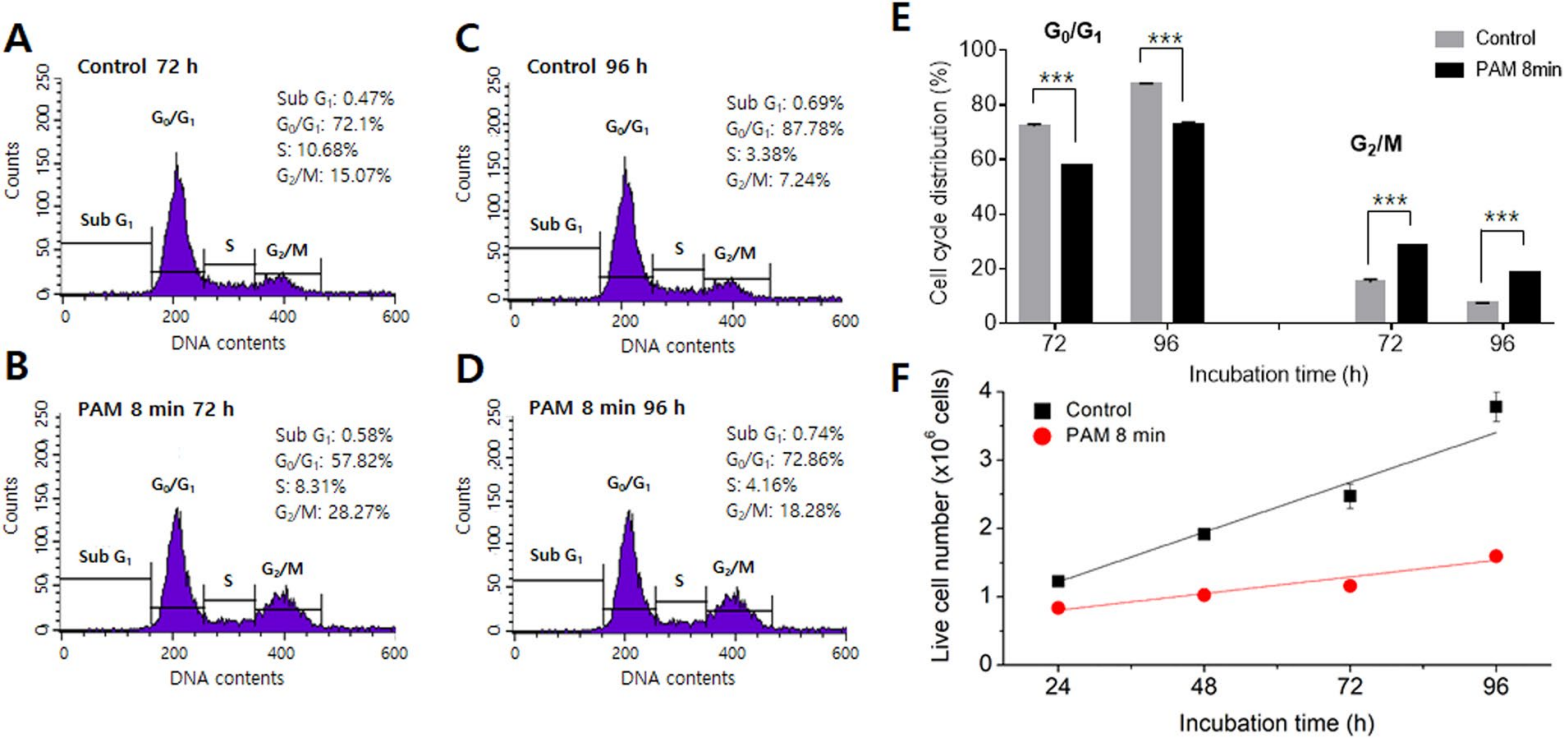
Cell cycle analysis of PAM-treated melanocytes. The cells were treated with PAM at a fixed dose of 8 min and incubated for an extended period. Cells were incubated in PAM for 48 h and then incubated in non-treated medium for an additional 48 h, for a total of 96 h. The cell cycles of the control and PAM-treated cells were analyzed after (**A**,**B**) 72 and (**C**,**D**) 96 h. (**C**) The changes in G_0_/G_1_ and G_2_/M phases of PAM-treated cells were compared with those of control cells. (**D**) The live cell number was analyzed as a function of incubation time.

To confirm the cell-cycle switching induced by PAM, melanocytes were treated with a PAM dose of 8 min and monitored for an extended period. In detail, the cells were exposed to a PAM dose of 8 min and incubated for 48 h. Then, the culture medium was changed, and the cells were incubated for an additional 24 or 48 h in new medium that had not been exposed to cold plasma. Figure 3A–D shows the results of cell cycle analysis of groups incubated for a total of 72 h (48 h in PAM + 24 h in untreated medium) and 96 h (48 h in PAM + 48 h in untreated medium). Compared to the control, apoptosis in the sub-G_1_ phase was slightly increased, but the difference was not significant in either group. According to changes in the S phase, DNA synthesis was inhibited in the group incubated for 72 h but increased in the group incubated for 96 h (Fig. 3B,D). That is, DNA synthesis appeared to gradually increase at a certain point following PAM treatment. As shown in Fig. 3E, the cell cycle distribution of control cells varied from group to group. In the G_0_/G_1_ phase, the distribution in the group incubated for 96 h was 13% higher than that in the group treated for 72 h. For the G_2_/M phase, the distribution in the 96 h group was 53% lower than that in the 72 h group. It is not clear why this difference occurred; however, the difference appeared to depend partially on the physiological state of the cells at the time of the experiment. In general, 0.3–2% of cells were in the sub-G_1_ phase, 70–74% in G_0_/G_1_, 15–20% in S, and 7–14% in G_2_/M^9,10^. Therefore, we considered only the differences between the control and experimental groups at the same time points. Compared to the control group, the cell cycle distribution in the G_0_/G_1_ phase was decreased by 20% and 17% in the 72 and 96 h incubated groups, respectively. This result clearly indicated that the cell cycle recovery progressed through reduced cell arrest and increased mitosis.

According to the results in Figs 2 and 3, the cells appeared to return to their original state through a recovery of cell cycle progression within 48 h after PAM treatment. However, because it took a considerable amount of time to fully recover to the normal state, many of the cells remained in a state of arrest. Therefore, even after 96 h, the proliferation rate of the PAM-treated cells (1.0 × 10^4^ cells/h) was lower than that of the control cells (3.0 × 10^4^ cells/h) (Fig. 3F).

### Melanogenesis activated by plasma-activated medium

**Figure 4:**
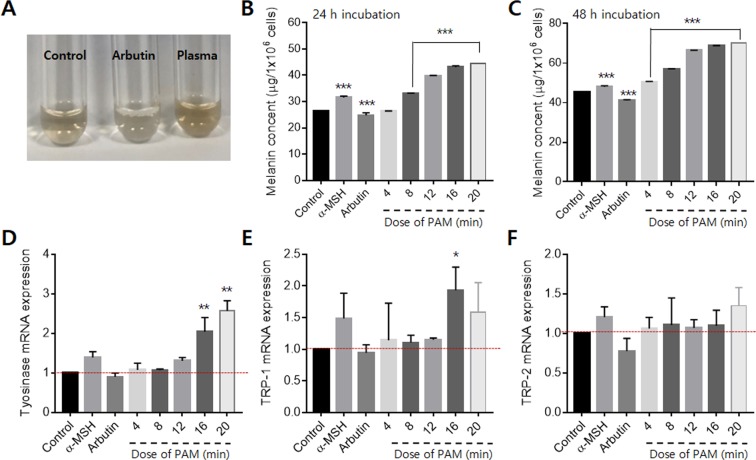
Melanin content analysis. (**A**) Color of cell lysates dissolved in NaOH, control, arbutin-treated, and PAM-treated cells. Changes in melanin contents depending on the treatment conditions and incubation times; i.e., (**B**) 24 and (**C**) 48 h. Changes in (**D**) tyrosinase, (**E**) TRP-1, and (F) TRP-2 depending on the treatment conditions.

We measured the intracellular melanin contents of melanocytes to assess the effects of PAM on melanogenesis. The plasma treatment protocol was used to investigate whether PAM could induce melanogenesis. Alpha-melanocyte-stimulating hormone (α-MSH), which is known to induce intracellular melanogenesis, was used as a positive control^11^. α-MSH is a class of peptide hormones that are produced by cells in the intermediate lobe of the pituitary gland and that stimulate the production and release of melanin by melanocytes. Arbutin, which is one of the most widely prescribed skin-lightening and depigmenting agents worldwide, was used as a negative control^12^. The color of cell lysates dissolved in NaOH clearly showed the melanogenesis effect of PAM (Fig. 4A). After 24 h of incubation, α-MSH increased the melanin content by 20% (Fig. 4B). The melanin content was decreased slightly, by 6%, by arbutin, and was increased by plasma in a dose-dependent manner. There was no change in melanin content at a PAM dose of 4 min, but the content increased at 8 min. At a PAM dose of 12 min, the melanin content increased more rapidly, becoming higher than that with α-MSH incubation. At 16 and 20 min, the increase in melanin slowed. After 48 h, it showed a similar change to that in melanin contents. Based on the highest value, which was observed at a PAM dose of 20 min, the content of melanin increased 1.7 times after 24 h and an additional 1.55 times after 48 h.

Melanogenesis in melanocytes is catalyzed by tyrosinase, TRP-1, and TRP-2. To investigate possible mechanisms responsible for the increase in melanin contents by PAM, the effects of PAM on tyrosinase, TRP-1, and TRP-2 activity were examined. The melanocytes were treated with PAM for 3 h, and then the activity of the three enzymes was measured. α-MSH induces intracellular tyrosinase activity, whereas arbutin inhibits tyrosinase activity^13^. The expression of tyrosinase was significantly increased in a dose-dependent manner by PAM (Fig. 4D). The increase in expression of tyrosinase by a high dose of PAM (16 and 20 min) was more pronounced than the increase by the tyrosinase inducer, α-MSH. After treatment with a high dose of PAM (20 min), the expression of tyrosinase increased up to 250% compared to the control cells. TRP-1 and TRP-2 did not increase in proportion to PAM dose, but all of the PAM-treated groups showed greater expression than the control group (Fig. 4E,F).

### ROS levels in melanocytes increased by plasma-activated medium

**Figure 5:**
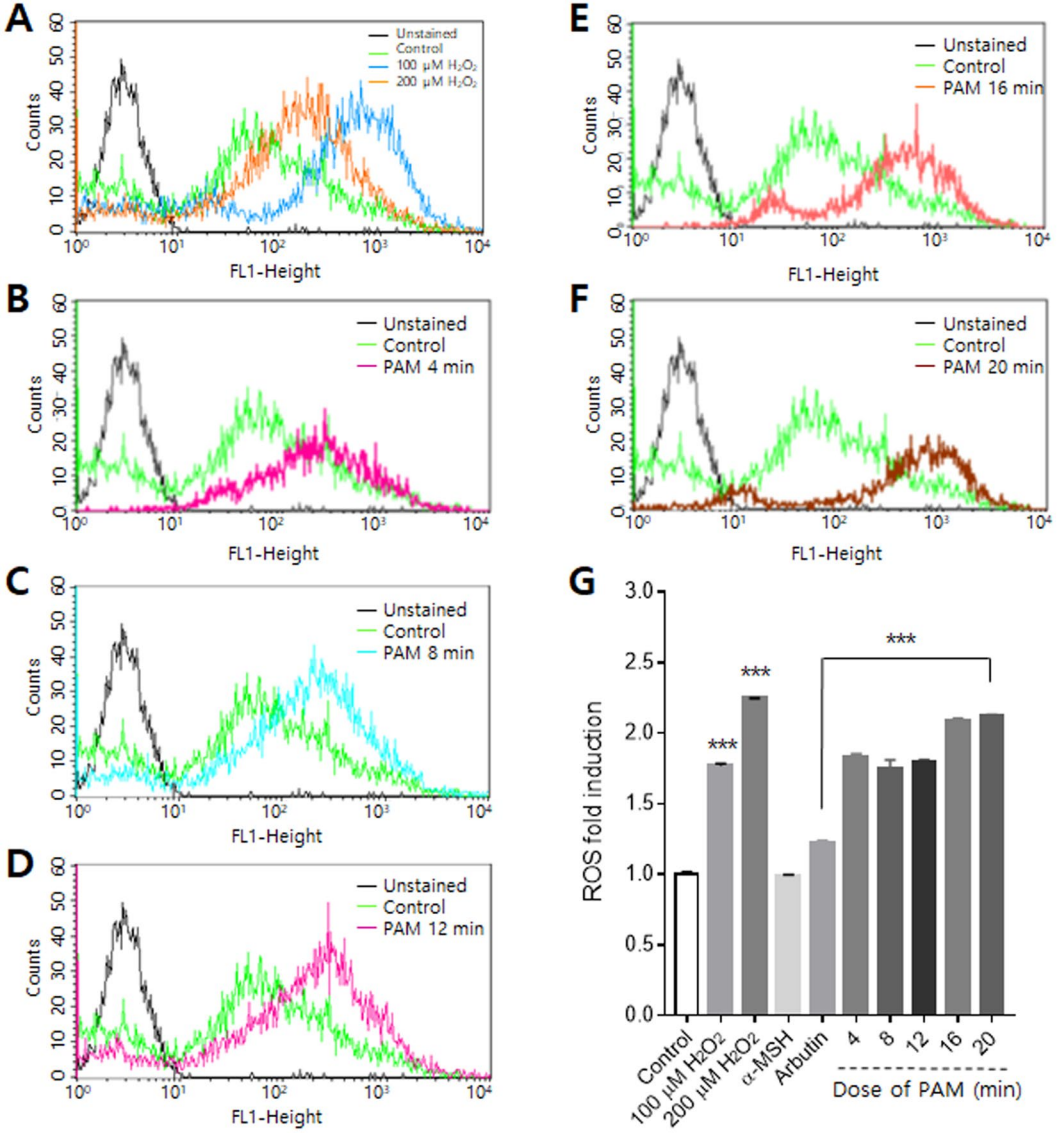
Intracellular ROS induced by PAM in melanocytes. (**A**) Changes in ROS level by H_2_O_2_ were investigated as a positive control. (**B**–**F**) ROS levels were increased by PAM treatment in all cases. (**G**) Comparison of ROS level changes according to the treatment conditions.

The increase in intracellular ROS in melanocytes induced by PAM was detected by 2′,7′-dichlorofluorescein diacetate (DCFH-DA) using flow cytometry. After a 3 h incubation of melanocytes in PAM, the intracellular ROS levels, including peroxide (H_2_O_2_), superoxide anion radical (O_2_), hydroxyl radical (OH), and singlet oxygen (^1^O_2_), in melanocytes were analyzed. The flow cytometry results are presented as the M2 percentage fluorescence (Fig. S2B), where M1, to the left, indicates cells that are not under stress and M2, to the right, indicates the percentage of cells with increased ROS production. We analyzed changes in M2 by PAM. The increase in ROS levels after exposure to H_2_O_2_ were investigated as a positive control, and the effects of α-MSH and arbutin were also studied. H_2_O_2_, itself a ROS, was used as a positive control. The exposure of melanocytes to H_2_O_2_ at 100 and 200 μM induced a significant increase in intracellular ROS levels of 1.8 and 2.2 fold, respectively, compared to untreated cells (Fig. 5A,G). α-MSH had no effect on ROS production. ROS levels were increased in all cells treated with PAM, as shown in Fig. 5B–F. The increase in ROS was not proportional to the PAM dose, and the increase by PAM was similar to that by H_2_O_2_ at 100 μM (Fig. 5G).

## Discussion

NAP plasma offers a new, non-invasive treatment method that uses highly reactive oxygen and nitrogen that can be effectively employed in various biological fields. NAP plasma is effective for immuno-proliferation, cell proliferation, protein stimulation, sterilization, and selective cancer apoptosis^14–16^. These functions of plasma are closely related to the generation of ROS. The amount of ROS produced depends on the energy generated by the plasma, and the variety of ROS depends on the type of gas used^17,18^. The functions of ROS in cells often contradict each other, depending on their contents. At low levels, ROS stimulate cell proliferation, angiogenesis, and metastasis^19,20^, whereas high levels of ROS can cause cellular damage, oxidative stress, and DNA damage^21,22^. There is no direct relationship between cell proliferation and ROS production. However, although high levels of ROS can lead to cell damage, a certain level of ROS is needed for the promotion of cell proliferation. According to a report by Zhang *et al*.^15^, L929 cell viability increased until the ROS content reached 6–7-fold that of the control; when ROS contents increased by more than 10-fold, cell viability decreased significantly. According to Chung *et al*.^17^, when the intracellular ROS contents of alveolar epithelial A549 cells were increased more than 3-fold by plasma treatment, the viability of cells was decreased to 87.54 ± 4.5% compared to the control. In our study, PAM induced only a 1.8–2.1-fold increase in ROS in melanocytes. This low level of ROS could not cause apoptosis; however, it inhibited cell proliferation.

The cell cycle is the fundamental process for the proliferation and growth of individual cells. Cell cycle progression is regulated by both internal and external factors. Internally, each stage of the cell cycle is mainly controlled by cyclin-dependent protein kinases (CDKs), including CDK1, −2, and −4^23^. In addition, several external factors influence the cell cycle, including nutrients, growth factors, and hormones, which affect the cells through intracellular signal transduction networks^24,25^. ROS have a crucial impact on the cell cycle, influencing cell cycle progression through phosphorylation and ubiquitination of CDKs^26^. Moreover, ROS inhibit the NF-κB pathway and modulate cyclins D, A, and p21^27,28^.

Cell cycle arrest occurs in two directions depending on the degree of stimulation, and the arrest may be transient or longer lasting. With weak stimulation, cell growth and division are inhibited by an increase in the population of G_0_/G_1_ cells. When the intensity of the stimulus reaches the limit of the cells, the population of G_0_/G_1_ cells is decreased, and apoptosis increases (sub-G_1_ phase). This is one of the working principles of anticancer drugs. A decrease in the populations of G_0_/G_1_ cells may cause an increase in phases other than sub-G_1_.

The cell cycle arrest observed in this study is thought to be related to ROS promoted by PAM. The impact of ROS on the cell cycle depends on the dose, and it ranges from transient growth arrest to permanent cell death, such as by apoptosis or necrosis. For comparison, ROS induced a transient multi-phase cell cycle arrest at the G_1_, S, and G_2_ phases of mouse fibroblasts, but had no effect on the M phase^28^. In another study, the accumulation of ROS induced arrest in G_2_/M phases and subsequent apoptosis^27^.

In the present study, PAM induced cell cycle arrest through regulation of the G_0_/G_1_ and G_2_/M phases, but it did not cause significant cell death. Furthermore, the cell cycle arrest was a transient phenomenon lasting 48 h or less, after which the cell cycle was slowly recovered. ROS generated by NAP plasma have a half-life in medium ranging from several hours to several days depending on temperature; however, the level of ROS decreases with time^7,8^. Therefore, the increased intracellular ROS level in melanocytes is also reduced with time, which may result in the recovery of cell cycle progression. Thus, we can summarize our findings as follows. PAM treatment at an appropriate concentration causes cell proliferation and cell cycle arrest, which can be recovered after a certain period of time. That is, the cell cycle can be turned on and off by NAP plasma without any intracellular toxicity.

**Figure 6:**
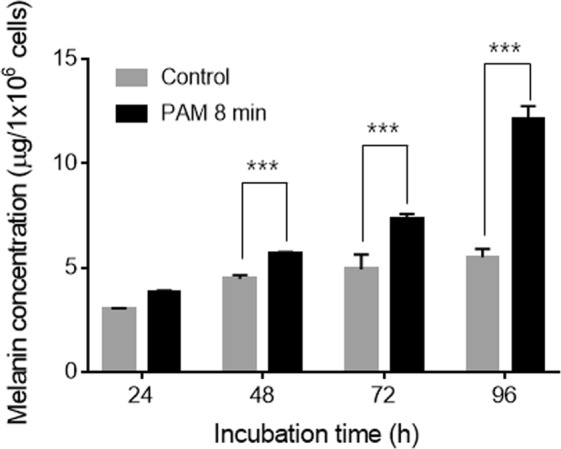
Comparison of changes in melanin concentration with incubation time in the control and PAM-treated groups.

Unexpectedly, the melanin contents were increased regardless of cell cycle arrest. We hypothesized that the melanin was increased by ROS to a certain level, and then decreased due to cell cycle arrest. However, the melanin content was proportional to the PAM dose, and the increase continued for 48 h after PAM treatment (Fig. 6). Melanin is synthesized in melanosomes, a small organelle synthesized in melanocytes. Melanogenesis comprises multiple processes: synthesis of tyrosinase, formation of a melanosome, and synthesis of melanin. There are three key enzymes involved in melanogenesis, tyrosinase, TRP-1, and TRP-2^29,30^. Tyrosinase, a glycoprotein located in the melanosome membrane, has a role in catalyzing the conversion of L-tyrosine into L-DOPA. Two proteins, TRP-1 and TRP-2, help to activate and stabilize tyrosinase and melanosome synthesis. The expression of all genes of tyrosinase, TRP-1, and TRP-2 was stimulated by PAM.

Regulation of the cell cycle has long been a focus of research. Our finding that the cell cycle can be switched on or off by PAM exposure provides insight into the characteristics of cancer cells and may facilitate the development of novel therapies. The treatment of cancer is hampered by our limited understanding of its recurrence, which plays a central role in disease progression and patient survival. Radiation and chemotherapy can induce irreversible cell-cycle arrest in cancer cells. However, this arrest of the cell cycle can be reversed by therapeutics, which may reflect recovery of the proliferative potential and metabolic activity of cancer cells^31^. Obtaining the desired skin tone and treating pigmentation abnormalities are popular cosmetic procedures. In Western culture, despite warnings of the deleterious effects of excessive exposure to sunlight or UV light, the number of tanning salons has expanded in the last few decades. In contrast, in Eastern culture, a light complexion is seen as equivalent to youth and beauty. Various methods and materials have been developed for bleaching hyperpigmented lesions or to achieve overall whitening, some of which are unsafe for use by humans^31–33^. We believe that modulation of melanogenesis by melanocytes will facilitate the control of skin tone without negative side effects.

In summary, we showed that cell cycle progression in normal human skin melanocytes could be switched on and off by PAM. Melanocytes treated with PAM exhibited cell cycle arrest for less than 48 h and then recovered without significant cytotoxic damage. Melanogenesis was accelerated by PAM through activation of tyrosinase, TRP-1, and TRP-2. Both transient cell cycle arrest and activated melanogenesis seem to be related to PAM-induced ROS.

## Materials and Methods

### Plasma-activated medium

**Figure 7:**
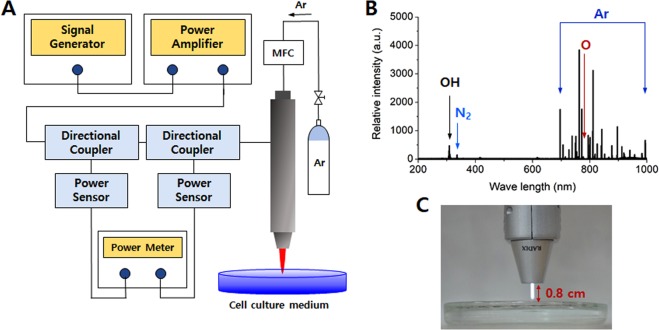
(**A**) Schematic representation of the NAP plasma generator. (**B**) Optical emission spectra of the NAP plasma jet in mode 3 during discharge at a wavelength of 200–1000 nm. (**C**) Experimental setup of the cold plasma jet used to prepare the PAM, which was taken by us.

We used a microwave plasma device (RADIX-0501; Medipl Co., Ltd., Gyeonggi-do, Korea) to generate NAP plasma. The plasma was generated by microwave power at atmospheric pressure using argon gas, and its temperature was less than 40 °C. The characteristics of the RADIX-0501 were as follows: microwave power of 2.0 W (Mode 1), 2.5 W (Mode 2), and 3.0 W (Mode 3); microwave accuracy of ±2.0%; gas pressure of 0–3 Bar; and gas flow controlled by 0–5 SLM. Figure 7 shows the emission spectra recorded by optical emission spectroscopy (DM750; Leica, Wetzlar, Germany) using the RADIX-0501 device operating in mode 3. The spectra emitted by excited atoms of the argon feed gas were between 690 and 900 nm. Peaks corresponding to N_2_ and O were measured at 337.2 and 777.4 nm, respectively. The OH band was detected between 307 and 309 nm. The distance between the end of plasma jet and the upper surface of the medium was fixed at 0.8 cm. A volume of 10 mL of medium was suspended in a 10 cm Petri dish and treated with plasma for 0–20 min. The PAM was applied to the melanocytes immediately.

### Cell culture and growth analysis

Normal human skin melanocytes were purchased from ATCC (Manassas, VA, USA). The melanocytes were grown in M254 medium (Gibco Cascade Biologics, Portland, OR, USA), supplemented with 1% (v/v) human melanocyte growth supplement (HMGS; Gibco Cascade Biologics, Portland, OR, USA), 100 U/mL penicillin, and 50 µg/mL streptomycin. The melanocytes were incubated in 10 cm cell-culture dishes (1 × 10^6^ cells/dish) with the PAM for 24 or 48 h. After incubation, the cells in each dish were harvested with trypsin-EDTA solution (JBI, Seoul, South Korea) and washed once with phosphate-buffered saline (PBS) containing 5% FBS. Then, the number of cells was counted using an ADAM-MC cell counter (NanoEnTeK, Seoul, South Korea).

### MTT assay

Cell viability was determined by 3-(4,5-dimethylthiazol-2-yl)-2,5-diphenyltetrazolium bromide (MTT) assay using 1 × 10^4^ cells/well in a 96-well plate. Purple formazan crystals were dissolved in dimethyl sulfoxide and transferred to a 96-well plate (200 μL/well), and the absorbance at 570 nm was determined using a microplate reader.

### WST-1 assay

Melanocytes were incubated in 96-well plates and incubated with PAM for 24 and 48 h. Next, the WST-1 cell proliferation reagent (Roche Applied Sciences, Mannheim, Germany) was added and the cells were incubated for 4 h. Finally, the absorbance of the samples at 450 nm was determined using a microplate reader at a reference wavelength of 650 nm.

### Lactate dehydrogenase (LDH) release assay

Cytotoxicity was assayed using the EZ-LDH Cytotoxicity Assay Kit (DoGen, Seoul, South Korea). In brief, melanocytes were incubated in PAM for 24 h, and 20 μL of supernatant from each well was transferred to a 96-well plate. Subsequently, 200 μL of LDH reaction mixture was added to each well and the plate was incubated at 37 °C for 30 min. The reaction was stopped by adding 20 μL of stop solution and mixing by gentle tapping. Next, the absorbance at 450 and 650 nm was measured. The cells were lysed in lysis buffer and the maximum LDH activity was determined according to the manufacturer’s instructions. Specific cytotoxicity was calculated as follows: Percentage lysis = (experimental − effector spontaneous − target spontaneous) ÷ (target maximum − target spontaneous) × 100.

### Cellular melanin contents

The determination of melanin content in melanocytes was performed as described in a previous report^6^. Sub-cultured melanocytes (1 × 10^6^ cells/well) were incubated in PAM for 24 and 48 h. The cells were treated with 1 μM α-MSH and 1 mM arbutin separately. The cells were dissolved in 1 N NaOH for 1 h at 60 °C. The absorbance was measured with a microplate reader (Spark 10 M; Tecan, Männedorf, Switzerland) at 37 °C at an absorption wavelength of 470 nm.

### Gene expression

For real-time PCR, the cells were seeded in 10 cm cell culture dishes (1.5 × 10^6^ cells /dish) for 72 h. The cells were treated with 1 μM α-MSH and 1 mM arbutin separately. The cells were treated with PAM exposed to plasma for 0, 4, 8, 12, 16, or 20 min. All treated cells were cultured in medium 254 with HMGS (Gibco Cascade Biologics, Portland, OR, USA) for 24 h. Total RNA was purified from the melanocytes using TRIzol reagent (Invitrogen, Carlsbad, CA, USA) following the manufacturer’s protocol.

### Reactive oxygen species production analysis

Melanocytes were seeded in 6 cm cell culture dishes (5 × 10^5^/dish) and allowed to attach for 24 h. The cells were treated with a-MSH (1 μM), arbutin (1 μM), and H_2_O_2_, (100 and 200 μM). PAM was provided to the melanocytes at varying doses (0, 4, 8, 12, 16, and 20 min). The cells were washed with PBS and incubated with 20 µM DCFH-DA (Sigma-Aldrich, St. Louis, MO, USA) diluted in medium 254 with HMGS for 30 min at 37 °C. Intracellular ROS levels were analyzed immediately by flow cytometry.

### Cell cycle analysis

Melanocytes were incubated in 10 cm cell culture dishes (1 × 10^6^ cells/dish) with PAM for 24 or 48 h at 37 °C in a humidified atmosphere containing 5% CO_2_. The untreated melanocytes were used to define the base level of apoptotic and dead cells. Cells were washed twice with PBS buffer, treated with 10 μg/mL RNase A, stained with 50 μg/mL propidium iodide (Sigma-Aldrich), and analyzed using a FACSCalibur flow cytometer (Becton Dickinson, Franklin Lakes, NJ, USA) and CellQuest 6.0 software.

### Statistics

All data are expressed as the means ± standard deviation (SD). Data were analyzed with two-tailed Student’s *t*-tests (SPSS ver. 13; SPSS Inc., Chicago, IL, USA). P-values < 0.01 were regarded as statistically significant.

## Supplementary information

Supplementary Figures
