# Supplementary material for: On–off switching of cell cycle and melanogenesis regulation of melanocytes by non-thermal atmospheric pressure plasma-activated medium
Source: Sci Rep. 2019 Sep 16;9:13400. doi: 10.1038/s41598-019-50041-2 (PMC6746696; doi:10.1038/s41598-019-50041-2)

On–off switching of cell cycle and melanogenesis regulation of melanocytes by non-thermal atmospheric pressure plasma-activated medium

Jin-Woo Lee[1], Se Jik Han[2,3], Hye Young Kang[4], Sung-Suk Wi[4], Min-HyungJung[5†], Kyung Sook Kim[3,6†]

[1]Medical Science Research Institute, Kyung Hee University Medical Center, Seoul, Korea
[2]Department of Biomedical Engineering, Graduate school, Kyung Hee University, Seoul, Korea
[3]Department of Biomedical Engineering, College of Medicine, Kyung Hee University, Seoul, Korea
[4]Research Laboratory, Medipl Co., Ltd, Gyeonggi-do, Korea
[5]Department of Obstetrics & Gynecology, School of Medicine, Kyung Hee University, Kyung Hee Medical Center, Seoul, Korea
[6]Healthcare Industry Research Institute, Kyung Hee University, Seoul, Republic of Korea

† These two authors equally contributed in this work

S1 Fig. Melanocyte viability as a function of plasma dose. The cells were incubated with PAM for 24 h, and then the MTT assay was conducted.

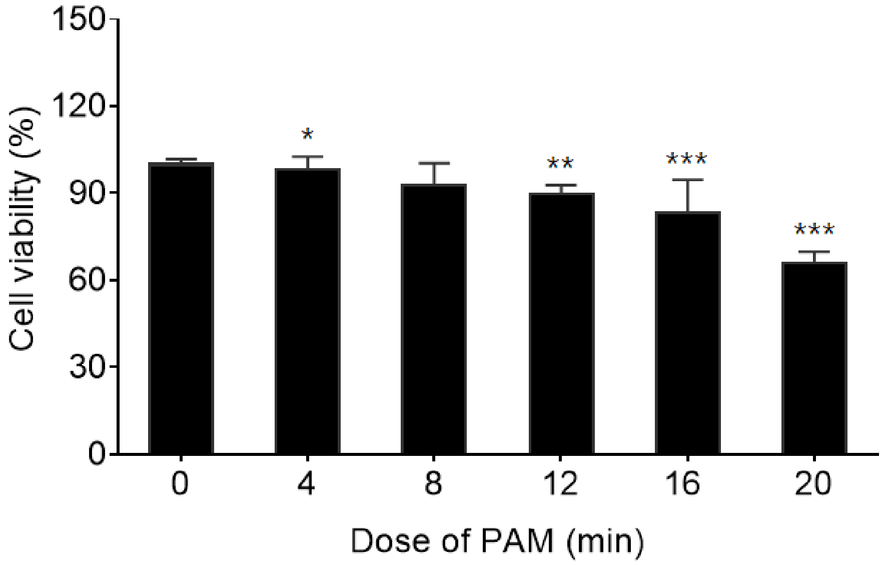

S2 Fig. (A) Intracellular ROS levels analyzed by flow cytometry. (B) M1 indicates cells not exposed to stress, and M2 indicates the percentage of cells with increased production of ROS. (C) ROS level as a function of PAM dose.

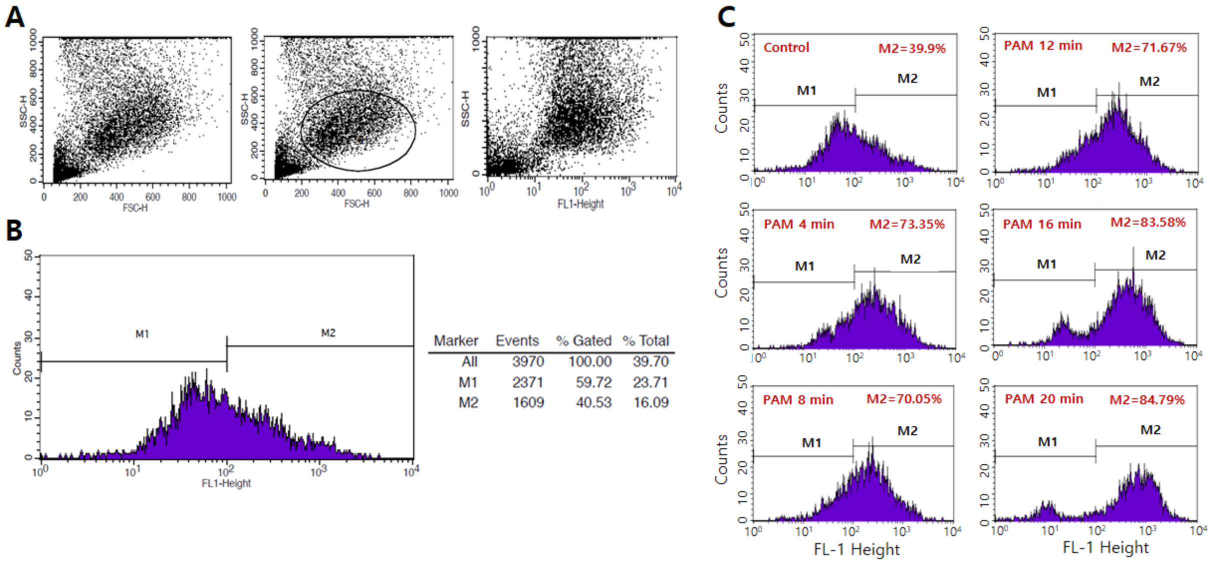

Supplement: Supplementary file 1 — Supplementary Figures [file 41598_2019_50041_MOESM1_ESM.pdf]
